# Efficacy and Risk Factors of Interferon-Gamma Release Assays among HIV-Positive Individuals

**DOI:** 10.3390/ijerph20054556

**Published:** 2023-03-04

**Authors:** Huifang Qin, Yiting Wang, Liwen Huang, Yan Huang, Jing Ye, Guijin Liang, Chongxing Zhou, Dabin Liang, Xiaoyan Liang, Yanlin Zhao, Mei Lin

**Affiliations:** 1 Guangxi Key Laboratory of Major Infectious Disease Prevention and Control and Biosafety Emergency Response, Nanning 530028, China; 2 Guangxi Zhuang Autonomous Region Center for Disease Control and Prevention, Nanning 530028, China; 3 Institute for Immunization and Prevention, Beijing Center for Disease Prevention and Control, Beijing 100013, China; 4 National Tuberculosis Reference Laboratory, Chinese Center for Disease Control and Prevention, Beijing 102206, China

**Keywords:** IGRA, latent TB infection, HIV

## Abstract

Latent tuberculosis is prevalent in HIV-infected people and has an impact on the progression of AIDS. The aim of this study is to match a more accurate IGRA method for the better detection of latent tuberculosis infection in HIV patients. All 2394 patients enrolled were tested using three IGRA methods. The positive rate consistency of pairwise comparison and risk factors were analyzed. Receiver operator characteristic (ROC) curve analysis was applied to evaluate the diagnostic value of T-SPOTTB. The positive rates of the three methods were statistically different (*p* < 0.001). The CD4^+^ T cell number statistically impacted the QuantiFERON and Wan Tai tests after the analysis with univariate logistic regression, while no statistical difference was observed in T-SPOT.TB. Additionally, there was a better sensitivity and specificity of T-SPOT.TB if the positive cut-off value of ESAT-6 and CFP-10 was 4.5 and 5.5, respectively. This study provides an insight into the IGRA methods and demonstrated that the positive response detected via QuantiFERON declined with decreased CD4^+^ T cells in the HIV-infected population; T-SPOT.TB functions independently of the CD4^+^ T cell level and Wan Tai was affected in some cases. This will be useful in the diagnosis of LTBI in the HIV-infected population, which will be a key step toward TB elimination in China.

## 1. Introduction

Tuberculosis (TB) is the only respiratory communicable disease among the top 10 causes of human death. *Mycobacterium tuberculosis*, the notorious pathogen of TB, infected about 10.0 million people globally in 2019 according to the World Health Organization (WHO) [1]. Additionally, people with immunodeficiency are more vulnerable to *M. tuberculosis*. The leading cause of death in HIV patients is co-infection with *M. tuberculosis* [2,3]. It was estimated that about 20.8 thousand HIV-positive people died of TB in 2019. Additionally, the rate of undiagnosed TB among HIV patients has been underestimated drastically, highlighting the need to focus on HIV-associated TB immediately [4].

Active TB was typically obtained from direct exposure to *M. tuberculosis* or the progress of the latent infection of TB. Additionally, one of the impact factors for progression was the immune status of individuals. Latent TB was hard to diagnose due to the absence of an accurate gold standard test [5]. Before IGRA was developed, the tuberculin skin test (TST) was widely used in epidemiological screening, as a result of its low cost and ease of operation [6]. However, the assay is criticized for its reduced sensitivity and specificity, and for cross-reaction to non-mycobacterium tuberculous proteins and the Bacilli Calmette–Guérin (BCG) vaccine [7]. Moreover, the TST performed poorly in HIV/TB co-infected patients [8].

Over the preceding decades, research has resulted in the development of the interferon-gamma release assay (IGRA), with the principle that T-cells of individuals who have acquired TB infection respond to re-stimulation with *M. tuberculosis*-specific antigens by secreting interferon-gamma (IFN-γ) [5]. In addition, IGRA was proven to be more effective in the diagnosis of latent TB than TST among diverse populations [9]. T-SPOT is an enzyme-linked immunospot assay measuring immune response by counting T cells in PBMC that can secrete the antigen-specific IFN-γ. QuantiFERON, a whole-blood ELISA method, measured the IFN-γ level secreted by specific T cells which were stimulated by early secreted antigenic target-6 (ESAT-6) and culture filtrate protein-10 (CFP-10) [10]. Wan Tai follows a similar basic approach as QuantiFERON; however, the antigen used in this assay was a recombinant ‘fusion’ protein of ESAT-6 and CFP-10. In China, all the three assays were applied in different studies [6,11,12], and there is a concern about whether the prominent IGRA assays endorsed by WHO for diagnosis of latent TB disease in HIV-infected populations were reproducible. We conducted this study to find a more precise IGRA method for detecting latent tuberculosis infection in HIV patients.

## 2. Materials and Methods

### 2.1. Study Population

From October 2019 to June 2021, a total of 2394 HIV/AIDS patients with no history of anti-tuberculosis treatment from 5 municipal and county-level medical institutions in Nanning, Liuzhou, Laibin, Chongzuo, and Lingshan in Guangxi province were enrolled. The median age of the participants was 48. The percentage of male patients was 61.1%. Peripheral blood samples were collected in lithium heparin anticoagulation vacuum tubes. The 3 IGRA methods were implemented to detect all of the samples.

Inclusion criteria were as follows: HIV-infected persons/patients included in the survey had to be newly registered or were previous cases currently available for follow-up at the local HIV prevention facility; since their HIV infection was confirmed, they had not taken anti-tuberculosis drugs; informed consent was also provided to participate in the survey. Exclusion criteria were as follows: patients with a previous history of tuberculosis or the use of anti-tuberculosis drugs; those who are not willing to sign the informed consent form; those who have serious illnesses that prevent them from participating in the survey were excluded from the research.

This study was approved by the Ethics Committee of the Institute for Tuberculosis Control and Prevention, Guangxi Center for Disease Control and Prevention (Nanning, China). Patients were included in this research only after written informed consent was received from the patient or their parent/guardian if the patient was <18 years of age.

### 2.2. Laboratory Tests

#### 2.2.1. Performing of the T-SPOT.TB Test

The T-SPOT.TB test (Oxford Immunotec Ltd., Abingdon, UK) was performed according to the instructions of the manufacturer. In brief, 5 mL of lithium heparin anticoagulated whole blood was used to complete cell separation using 1640 culture medium, Ficoll lymphocyte separation medium, and cell culture medium. Isolated cells were diluted and counted with a biological microscope or cell counter. For each test sample, one blank control and one control for judging cell activity were set up before adding samples to the culture plate and incubation. After the culture plate was washed and spots formed, the number of spots was counted with a magnifying glass or biological microscope, and the results were judged according to the number of spots.

#### 2.2.2. Performing the QuantiFERON and Wan Tai Test

The procedures of the QuantiFERON and Wan Tai tests were consistent with manufacturers’ guidelines. A total of 1 mL of lithium heparin anticoagulated whole blood samples was dispensed into a nil control tube, TB antigen tube, and mitogen control tube (T, N, and P tube in Wan Tai) for 16 h. The TB antigen tube and T tube contained ESAT-6 and CFP-10 antigens, the nil tube, and N tube were used for the negative control, while the mitogen tube and P tube with phytohemagglutinin were used for positive control. After the whole blood was injected into the culture tube, it was inverted and mixed well, moved to a 36 ± 1 °C incubator, and incubated for 22–24 h with the culture tube upright during the process. After incubation, plasma was separated by centrifuging the culture tube at 3000 rpm for 10 min for the ELISA assay to detect IFN-γ.

### 2.3. Statistical Analysis

Univariate logistic regression was used to evaluate the effect of age, gender, CD4^+^ T cell, HIV diagnosis year, and HAART on different IGRA detecting methods. Chi-square analysis was performed to evaluate the pairwise comparison between 3 methods stratified by CD4^+^ T cell. A *p*-value of <0.05 was considered statistically significant. All statistical data were analyzed using SPSS v.23 (SPSS Inc., Chicago, IL, USA).

## 3. Results

### 3.1. Performance of the Three Methods

There were 2394 cases in total. The positive cases detected using T-SPOT.TB and Wan Tai were 405 and 369, respectively. QuantiFERON detected 457 positive cases, a little more than the other methods.

**Figure 1:**
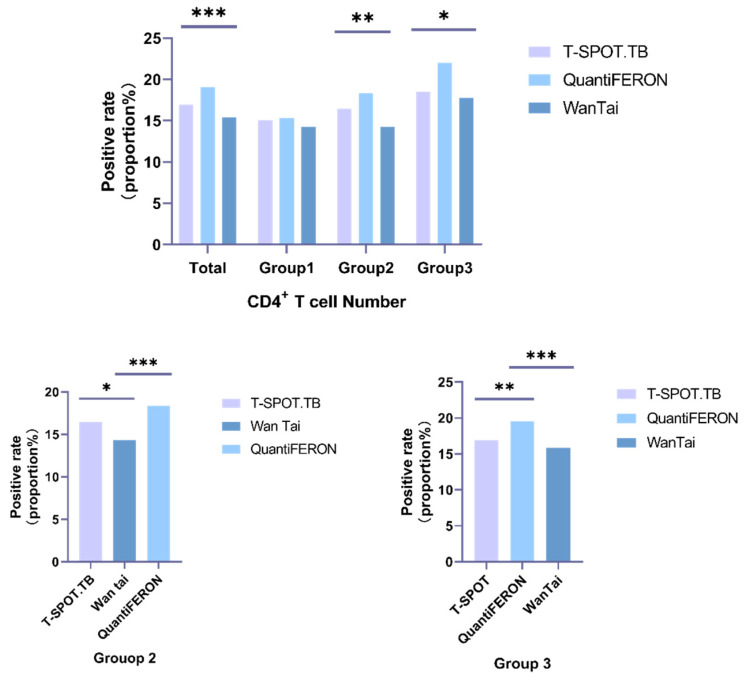
Positive rate of the three IGRA methods and pairwise comparison stratified by different groups. Group 1: CD4^+^ T cell number < 200/μL; Group 2: CD4^+^ T cell number 200–500/μL; Group 3: CD4^+^ T cell number > 500/μL; *: *p* ≤ 0.05; **: *p* ≤ 0.01; ***: *p* ≤ 0.001.

The positive rate of the three IGRA methods is shown in Figure 1. The positive rate for T-SPOT.TB, QuantiFERON, and Wan Tai in all HIV-infected patients was 16.92% (405/2394), 19.09% (457/2394), and 15.41% (369/2394), respectively. Statistical differences were observed among the three groups.

### 3.2. The Consistency of T-SPOT.TB QuantiFERON and Wan Tai

The pairwise comparison of positive results is shown in Table 1. The consistency rate between T-SPOT.TB and QuantiFERON was 81.20% (1944/2394). Compared to QuantiFERON, T-SPOT.TB had a higher agreement rate with Wan Tai, which was 85.71% (2052/2394). Additionally, due to the same detection theory, QuantiFERON was also more consistent with Wan Tai with a consistency rate of 82.79% (1982/2394). The total coincidence rate of the three methods was 74.85% (1792/2394).

### 3.3. Analysis of Impact Factors for Three Tests after Logistic Regression

Univariate logistic regression was used to evaluate the effect of age, gender, CD4^+^ T cell number, HIV diagnosis year, and highly active antiretroviral therapy history (HAART) on different IGRA detecting assays. Although there was no obvious difference when considering age, gender, HIV diagnosis year, and HAART history, the CD4^+^ T cell number statistically impacted the detecting methods. Thus, a chi-square analysis was performed to evaluate the pairwise correlation between three methods stratified by CD4^+^ T cell number.

The number of cases divided into three groups was, respectively, 385, 1210, and 799. Cases in group 2 were the most, with the the proportion being 50.54% (1210/2394); 33.38% (799/2394) of cases were in group 3, and about 16.08% (385/2394) participants had a CD4^+^ T cell count of <200/μL (group 1).

As shown in Figure 1, the positive rates of T-SPOT.TB, QuantiFERON, and Wan Tai in group 1 were 15.06%, 15.32%, and 14.29%, respectively. There was no statistical difference between the three methods (*p* = 0.855). While in group 2, the positive rates were 16.44%, 18.35%, and 14.21%, respectively, and a statistical difference was observed (*p* = 0.002). The rates then rose to 18.52%, 22.03%, and 17.77% in group 3. There were statistical differences between the three methods (*p* = 0.011).

In all groups, QuantiFERON detected the highest number of positive results, followed by T-SPOT.TB. Additionally, the positive rates increased obviously with the CD4^+^ T cell count rising when tested with T-SPOT.TB or QuantiFERON.

**Figure 2:**
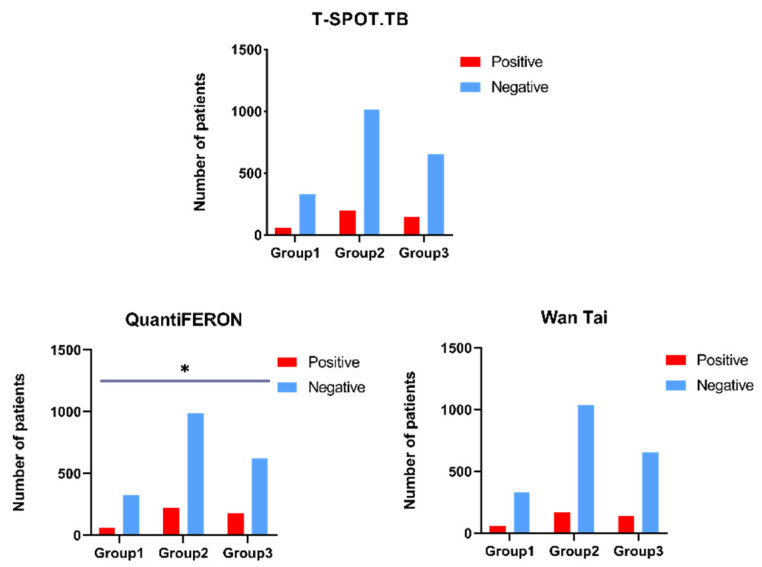
The comparison of different groups with different IGRA assays. Group 1: CD4^+^ T cell number of <200/μL; Group 2: CD4^+^ T cell number of 200–500/μL; Group 3: CD4^+^ T cell number of >500/μL; *: *p* ≤ 0.05.

Comparing three different CD4^+^ T cell groups with distinct detecting methods, the result showed that QuantiFERON had statistical differences among the three groups (*p* = 0.015), while using T-SPOT.TB (*p* = 0.273) and Wan Tai, there was no statistically significant difference observed (*p* = 0.077) (Figure 2).

### 3.4. Evaluation of T-SPOT.TB and ROC Analysis

**Figure 3:**
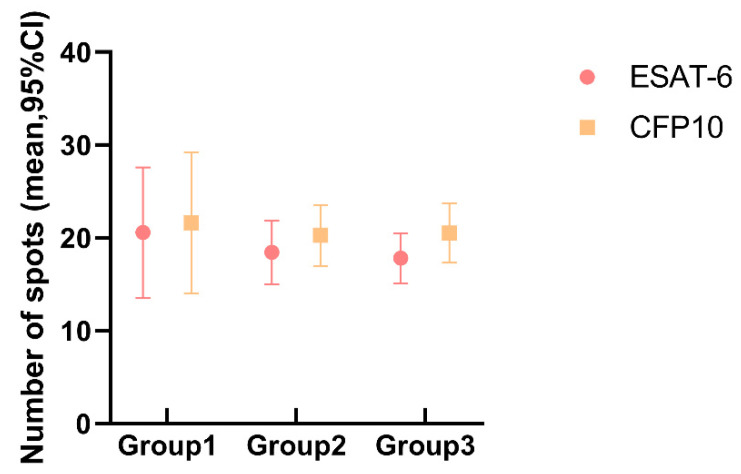
The spot number of ESAT-6 and CFP-10 in different CD4^+^ T cell groups.

The spot distribution of T-SPOT.TB in different subgroups is shown in Figure 3. There is no statistical difference between the three subgroups, with Kruskal–Wallis test *p*-values of 0.647 and 0.776 for the two antigens, respectively.

**Figure 4:**
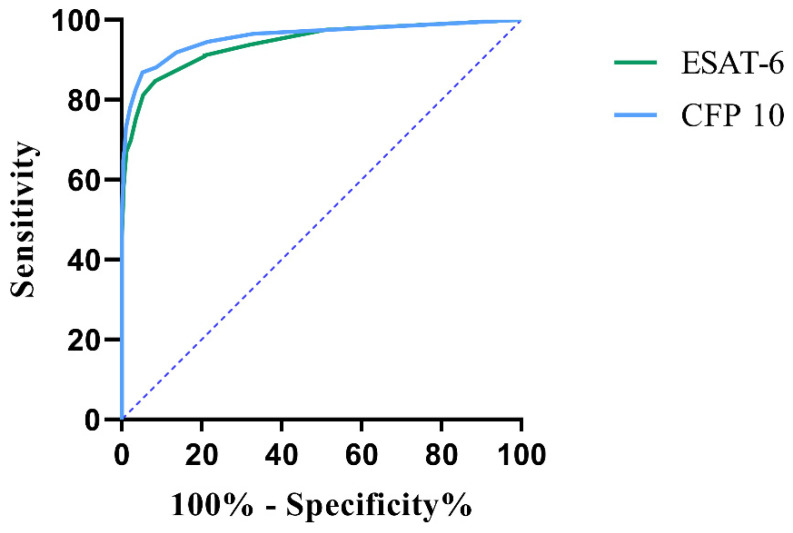
Receiver operating characteristic (ROC) curve analysis of T-SPOT.TB test.

The area under the ROC curve (AUROC) was detected to evaluate the diagnostic value. There would be a better sensitivity and specificity if the positive cut-off value of ESAT-6 was 4.5 (AUROC = 0.94, 95% CI 0.9266–0.9558) and that of CFP-10 was 5.5 (AUROC = 0.96, 95% CI 0.9437–0.9701). This specific cut-off value for HIV-TB coinfected patients may clinically improve diagnostic accuracy (Figure 4).

## 4. Discussion

The positive rate of HIV patients in this study was 15.41–16.92% using different detecting methods, lower than that detected by Ngai Sze Wong et al. [13], who detected 32% of LTBI patients in HIV-infected patients from tuberculin skin testing (tuberculin skin testing, TST) or IGRA. This inconsistency may be due to the different detection methods: TST was reported to be different from IGRA methods in several pieces of research [5,6,14].

In this study, we demonstrated that the performance of the three detecting methods for HIV patients varied based on different HIV infection stages. The positive rate of QuantiFERON was statistically higher than the others, which is in accordance with earlier research [11,15,16]. This may be due to the different detection theories: QuantiFERON is an ELISA assay developed using an early secretory antigenic target (ESAT-6) and the 10-kDa culture filtrate protein (CFP-10) to stimulate T cells from whole blood in vivo to quantify specific IFN-γ [5]. As is well known, interferon-gamma is secreted by a wide variety of cells under a range of inflammatory conditions, including CD4^+^ T helper cell type 1 (Th1) lymphocytes, CD8+ cytotoxic lymphocytes, NK cells, B cells, NKT cells, and professional antigen-presenting cells (APCs), etc. [17,18,19]. Additionally, other non-PBMC cells in the blood such as neutrophils can also affect the secretion of IFN-γ [20]. On the other hand, T-SPOT.TB is an ELISPOT assay which detects IFN-γ-secreted effective T cells in PBMC rather than whole blood, and was less susceptible to non-effective T cells. Another possible reason for the lower positive rate of T-SPOT.TB could be a longer processing time of samples [15]. Further study was needed to evaluate this impact.

We also analyzed the impact factors for three tests using logistic regression, and variations such as age, gender, CD4^+^ T cell number, HIV diagnosis year, and HAART history were considered. A statistically significant difference is observed in QuantiFERON, especially in the subgroup with a CD4^+^ T cell number of ≤500. In addition, this study and others [13,21] showed that the positive rate of QuantiFERON slightly declined with a decreased CD4^+^ T cell count. CD4^+^ T cell plays an important role both in the pathogenesis of HIV and the defense of tuberculosis. The deficiency of CD4^+^ T cells could be attributed to the immunosuppression of antimycobacterial immunity during HIV infection, which has been demonstrated to decrease M. tuberculosis-specific IFN-γ release in another research [22,23].

In line with previous results, we observed that age, gender, and HAART history did not affect the positive rate of any method [24,25]. The HIV diagnosis year also did not affect the test results in our study.

Wan Tai is recommended by WHO to be one of the alternatives when detecting LTBI, based on published data analysis and a comprehensive consideration of indicators such as sensitivity and specificity. In our study, Wantai was affected by CD4^+^ T cell counts, when CD4^+^ T cells ranged from 200/μL to 500/μL (*p* = 0.032, Table 2). However, when comparing the results of Wantai and QuantiFERON in patients with CD4^+^ T cells of >200/μL, statistical differences were observed (Figure 1). One possible reason was that a different antigen extraction had been processed and more data from various population groups were needed to compare those two products. Lin Chuan Wang et al. has shown that there was no statistically significant difference between the positive rate of T-SPOT.TB and Wan tai [11]; however, the specificity of T-SPOT.TB was higher than Wan Tai. In our study, we demonstrated the statistical differences between Wan Tai and T-SPOT.TB when the CD4^+^ T cell count ranged from 200/μL to 500/μL, with a *p*-value of 0.046. The discrepancy occurrence may be due to the different populations involved in the two studies, and whether paired samples were applied.

From our perspective, T-SPOT.TB is not affected by CD4^+^ T cell number, maintaining a relatively stable detection efficiency, just the same as in a previous study [25]. The number of spot-forming cells (SFCs) reactive to ESAT-6 and CFP-10 detected by T-SPOT.TB was analyzed in different CD4^+^ T cell count groups. No statistical difference was observed between subgroups, which was similar to a previous study [26]. However, this finding is contrary to an earlier study which has suggested that the number of SFCs reactive with ESAT-6 and CFP-10 positively correlated with the number of circulating CD4^+^ T-cells [25], and at the same time, there was no statistical difference between CD4^+^ T cells and the T-SPOT.TB positive rates. The reason for the discrepancy may be that patients in our study were HIV-infected patients rather than active TB patients with HIV infections. ROC curves were used to evaluate the T-SPOT.TB test and data showed that the AUC for ESAT-6 and CFP-10 were 0.94 and 0.96, respectively, which was higher than those reported by Lin Chuan Wang et al., Janssens et al., and Ling et al. [12,27,28], suggesting a higher accuracy of T-SPOT.TB with a cut-off of 4.5 and 5.5 for ESAT-6 and CFP-10, respectively.

The limitations of our study need to be addressed. While we performed the comparison of the three recommended IGRA methods to evaluate their effectiveness among HIV-infected patients, HIV type was not considered. Farba Karam et al. have demonstrated that the positive response to ELISPOT assay was associated with HIV-1 strain [29], which should be noticed in further study considering the epidemiological and biological differences of the HIV type. Additionally, given there is no gold standard for LTBI diagnosis, the performance of the three IGRA methods for the diagnosis of latent TB infection cannot be evaluated. Though there is an increasing number of studies on the appraisal of IGRA methods, more longitudinal studies on the evaluation of IGRA for the diagnostic value of LTBI are needed.

## 5. Conclusions

Latent TB infection is a severe cause of death for HIV patients. Effective diagnosis and timely treatment are crucial to co-infected patients. We demonstrated that the positive rate of TB infection in HIV patients in Guangxi Province was about 15.41–16.92%, as detected by various IGRA methods. Due to the high infection rate of TB among HIV patients, routine tuberculosis screening programs should be considered among the HIV-infected population to prevent tuberculosis transmission. Additionally, to achieve a more accurate diagnosis, different testing methods should be used depending on the HIV patients’ stage of infection. The positive response detected by QuantiFERON slightly declined with a decreased CD4^+^ T cell count. However, T-SPOT.TB functions independently of the CD4^+^ T cell number level, maintaining a relatively stable detection efficiency. For patients in the terminal stage of HIV infection, T-SPOT.TB would be a better choice in the diagnosis of TB latent infection. Therefore, our data suggested that a different result interpretation criteria and cut-off should be constructed to distinguish latent TB-infected HIV patients. Research with larger sample sizes from other provinces or countries is required to optimize the current criteria and construct a more refined interpretation of the results.

## Figures and Tables

**Table 1:** The consistency between T-SPOT.TB, QuantiFERON, and WanTai.

	Consistant Positive (n)	Consistant Negative (n)	Inconsistant Number (n)	Total (n)	Consistent Rate (%)
T-SPOT.TB vs. QuantiFERON	206	1738	450	2394	81.20
T-SPOT.TB vs. WanTai	216	1836	342	2394	85.71
QuantiFERON vs. WanTai	207	1775	412	2394	82.79

**Table 2:** Univariate analysis of factors associated with the three IGRA methods.

Factors	No. of Cases (%)	T-SPOT.TB		QuantiFERON		Wan Tai	
		*p*-Value	OR (95% CI)	*p*-Value	OR (95% CI)	*p*-Value	OR (95% CI)
Age group							
<30	160 (6.6)		Ref.		Ref.		Ref.
30–45	844 (34.7)	0.195	0.727 (0.449–1.178)	0.692	1.090 (0.711–1.673)	0.059	0.596 (0.348–1.020)
46–60	837 (34.4)	0.089	0.785 (0.593–1.037)	0.686	0.946 (0.724–1.237)	0.238	0.84 (0.630–1.122)
>60	553 (22.8)	0.146	0.813 (0.616–1.075)	0.129	0.809 (0.615–1.064)	0.217	0.833 (0.624–1.113)
Gender							
Male	1484 (61.1)		Ref.		Ref.		Ref.
Female	910 (37.4)	0.504	0.927 (0.743–1.157)	0.058	0.813 (0.657–1.007)	0.932	1.010 (0.804–1.269)
CD4^+^ T cell number							
<200/μL	385 (15.8)	0.142	0.780 (0.560–1.087)	0.007 *	0.641 (0.463–0.886)	0.132	0.771 (0.550–1.081)
200–500/μL	1210 (49.8)	0.228	0.866 (0.685–1.095)	0.043 *	0.795 (0.637–0.993)	0.032 *	0.767 (0.601–0.977)
>500/μL	799 (32.9)		Ref.		Ref.		Ref.
HIV diagnosis year							
1–5	1045 (43.0)		Ref.		Ref.		Ref.
6–10	944 (38.8)	0.953	1.046 (0.230–4.763)	0.728	0.794 (0.216–2.911)	0.956	0.958 (0.210–4.363)
11–15	391 (16.1)	0.807	1.208 (0.265–5.500)	0.669	0.753 (0.205–2.766)	0.977	1.023 (0.224–4.661)
>15	13 (0.5)	0.887	1.117 (0.242–5.57)	0.800	0.844 (0.227–3.139)	0.925	1.076 (0.233–4.973)
HAART							
treated	2363 (97.2)	0.189	0.581 (0.258–1.307)	0.970	0.983 (0.401–2.410)	0.542	0.7756 (0.308–1.856)
untreated	31 (1.3)		Ref.		Ref.		Ref.

Abbreviation: HAART, highly active antiretroviral therapy; OR, odds ratio; CI, confidence interval; * Statistically significant (*p* < 0.05).

## Data Availability

Data not available due to ethical restrictions.

## References

[1] Chakaya J., Khan M., Ntoumi F., Aklillu E., Fatima R., Mwaba P., Kapata N., Mfinanga S., Hasnain S.E., Katoto P. (2021). Global Tuberculosis Report 2020—Reflections on the Global TB burden, treatment and prevention efforts. Int. J. Infect. Dis..

[2] Bell L.C.K., Noursadeghi M. (2018). Pathogenesis of HIV-1 and Mycobacterium tuberculosis co-infection. Nat. Rev. Microbiol..

[3] Bates I., Fenton C., Gruber J., Lalloo D., Medina Lara A., Squire S.B., Theobald S., Thomson R., Tolhurst R. (2004). Vulnerability to malaria, tuberculosis, and HIV/AIDS infection and disease. Part 1: Determinants operating at individual and household level. Lancet Infect. Dis..

[4] Gupta R.K., Lucas S.B., Fielding K.L., Lawn S.D. (2015). Prevalence of tuberculosis in post-mortem studies of HIV-infected adults and children in resource-limited settings: A systematic review and meta-analysis. AIDS.

[5] Carranza C., Pedraza-Sanchez S., de Oyarzabal-Mendez E., Torres M. (2020). Diagnosis for Latent Tuberculosis Infection: New Alternatives. Front. Immunol..

[6] Gao L., Lu W., Bai L., Wang X., Xu J., Catanzaro A., Cardenas V., Li X., Yang Y., Du J. (2015). Latent tuberculosis infection in rural China: Baseline results of a population-based, multicentre, prospective cohort study. Lancet Infect. Dis..

[7] Wang L., Turner M.O., Elwood R.K., Schulzer M., FitzGerald J.M. (2002). A meta-analysis of the effect of Bacille Calmette Guerin vaccination on tuberculin skin test measurements. Thorax.

[8] Keramat F., Bagheri Delavar B., Zamani A., Poorolajal J., Lajevardi E., Saadatmand A. (2020). Comparison of Quantiferon-TB Gold and TST tests in the diagnosis of latent tuberculosis infection among HIV infected patients in Hamadan, west of Iran. J. Infect. Dev. Ctries..

[9] Lawn S.D., Bangani N., Vogt M., Bekker L.G., Badri M., Ntobongwana M., Dockrell H.M., Wilkinson R.J., Wood R. (2007). Utility of interferon-gamma ELISPOT assay responses in highly tuberculosis-exposed patients with advanced HIV infection in South Africa. BMC Infect. Dis..

[10] Mori T., Sakatani M., Yamagishi F., Takashima T., Kawabe Y., Nagao K., Shigeto E., Harada N., Mitarai S., Okada M. (2004). Specific detection of tuberculosis infection: An interferon-gamma-based assay using new antigens. Am. J. Respir. Crit. Care Med..

[11] Wang L., Tian X.D., Yu Y., Chen W. (2018). Evaluation of the performance of two tuberculosis interferon gamma release assays (IGRA-ELISA and T-SPOT.TB) for diagnosing Mycobacterium tuberculosis infection. Clin. Chim. Acta.

[12] Wang L., Yu Y., Chen W., Feng J., Wang J., Zhao H., Ma L., Yang B., Ma Y., Dang P. (2015). Evaluation of the characteristics of the enzyme-linked immunospot assay for diagnosis of active tuberculosis in China. Clin. Vaccine Immunol..

[13] Wong N.S., Leung C.C., Chan K.C.W., Chan W.K., Lin A.W.C., Lee S.S. (2019). A longitudinal study on latent TB infection screening and its association with TB incidence in HIV patients. Sci. Rep..

[14] Surve S., Bhor V., Naukariya K., Begum S., Munne K., Tipre P., Sutar N., Jaiswal A., Bhonde G., Chauhan S. (2021). Discordance between TST and QFT-TBGold Plus for Latent Tuberculosis Screening among Under-Five Children: An Interim Analysis. J. Trop. Pediatr..

[15] Venkatappa T.K., Punnoose R., Katz D.J., Higgins M.P., Banaei N., Graviss E.A., Belknap R.W., Ho C.S. (2019). Comparing QuantiFERON-TB Gold Plus with Other Tests To Diagnose Mycobacterium tuberculosis Infection. J. Clin. Microbiol..

[16] Zhang H., Xin H., Wang D., Pan S., Liu Z., Cao X., Wang J., Li X., Feng B., Li M. (2019). Serial testing of Mycobacterium tuberculosis infection in Chinese village doctors by QuantiFERON-TB Gold Plus, QuantiFERON-TB Gold in-Tube and T-SPOT.TB. J. Infect..

[17] Schroder K., Hertzog P.J., Ravasi T., Hume D.A. (2004). Interferon-gamma: An overview of signals, mechanisms and functions. J. Leukoc. Biol..

[18] Frucht D.M., Fukao T., Bogdan C., Schindler H., O’Shea J.J., Koyasu S. (2001). IFN-gamma production by antigen-presenting cells: Mechanisms emerge. Trends Immunol..

[19] Flaishon L., Hershkoviz R., Lantner F., Lider O., Alon R., Levo Y., Flavell R.A., Shachar I. (2000). Autocrine secretion of interferon gamma negatively regulates homing of immature B cells. J. Exp. Med..

[20] Denkers E.Y., Del Rio L., Bennouna S. (2003). Neutrophil production of IL-12 and other cytokines during microbial infection. Chem. Immunol. Allergy.

[21] Birku M., Desalegn G., Kassa G., Tsegaye A., Abebe M. (2020). Effect of pregnancy and HIV infection on detection of latent TB infection by Tuberculin Skin Test and QuantiFERON-TB Gold In-Tube assay among women living in a high TB and HIV burden setting. Int. J. Infect. Dis..

[22] Diedrich C.R., Flynn J.L. (2011). HIV-1/mycobacterium tuberculosis coinfection immunology: How does HIV-1 exacerbate tuberculosis?. Infect. Immun..

[23] Petruccioli E., Chiacchio T., Navarra A., Vanini V., Cuzzi G., Cimaglia C., Codecasa L.R., Pinnetti C., Riccardi N., Palmieri F. (2020). Effect of HIV-infection on QuantiFERON-plus accuracy in patients with active tuberculosis and latent infection. J. Infect..

[24] Telisinghe L., Amofa-Sekyi M., Maluzi K., Kaluba-Milimo D., Cheeba-Lengwe M., Chiwele K., Kosloff B., Floyd S., Bailey S.L., Ayles H. (2017). The sensitivity of the QuantiFERON((R))-TB Gold Plus assay in Zambian adults with active tuberculosis. Int. J. Tuberc. Lung Dis..

[25] Cai R., Chen J., Guan L., Sun M., Sun Y., Shen Y., Zhang R., Liu L., Lu H. (2014). Relationship between T-SPOT.TB responses and numbers of circulating CD4+ T-cells in HIV infected patients with active tuberculosis. Biosci. Trends.

[26] Leidl L., Mayanja-Kizza H., Sotgiu G., Baseke J., Ernst M., Hirsch C., Goletti D., Toossi Z., Lange C. (2010). Relationship of immunodiagnostic assays for tuberculosis and numbers of circulating CD4+ T-cells in HIV infection. Eur. Respir. J..

[27] Janssens J.P., Roux-Lombard P., Perneger T., Metzger M., Vivien R., Rochat T. (2007). Quantitative scoring of an interferon-gamma assay for differentiating active from latent tuberculosis. Eur. Respir. J..

[28] Ling D.I., Pai M., Davids V., Brunet L., Lenders L., Meldau R., Calligaro G., Allwood B., van Zyl-Smit R., Peter J. (2011). Are interferon-gamma release assays useful for diagnosing active tuberculosis in a high-burden setting?. Eur. Respir. J..

[29] Karam F., Mbow F., Fletcher H., Senghor C.S., Coulibaly K.D., LeFevre A.M., Ngom Gueye N.F., Dieye T., Sow P.S., Mboup S. (2008). Sensitivity of IFN-gamma release assay to detect latent tuberculosis infection is retained in HIV-infected patients but dependent on HIV/AIDS progression. PLoS ONE.

